# Supplementary material for: Curvature controlled defect dynamics in topological active nematics
Source: Sci Rep. 2017 Jul 12;7:5211. doi: 10.1038/s41598-017-05612-6 (PMC5507919; doi:10.1038/s41598-017-05612-6)
Supplement: Supplementary file 6 — supplementary information [file 41598_2017_5612_MOESM6_ESM.pdf]

# Curvature controlled defect dynamics in topological active nematics

Francesco Alaimo et al.

### Supporting information

We highlight the differences between the defect trajectories and their coarse-grained description by self-propelled particles. Figure 1 SI shows the trajectories on three different spheroidal ellipsoids, an oblate, a sphere and a prolate. The differences on the sphere have already been pointed out in the main text. On an oblate all particles are located at the waist and rotate and on a prolate we have again closed trajectories, which cover the poles and leave various parts around the waist empty.

All movies correspond to the snapshots shown in Figure 1, Figure 3 a), b) and Figure 4, showing the nematic order parameter $P$, defined in Section Numerical Methods, through the underlying color, the director field as black lines and highlighted defects. The same visualization is used for Figure 6 for the case $a/b = 1.1$ and $a/c = 6$.

- `video_figure1_back_and_front.mpeg`
- `video_figure3a_back_and_front.mpeg`
- `video_figure3b_back_and_front.mpeg`
- `video_figure4_back_and_front.mpeg`

- video_figure6_back_and_front.mpeg

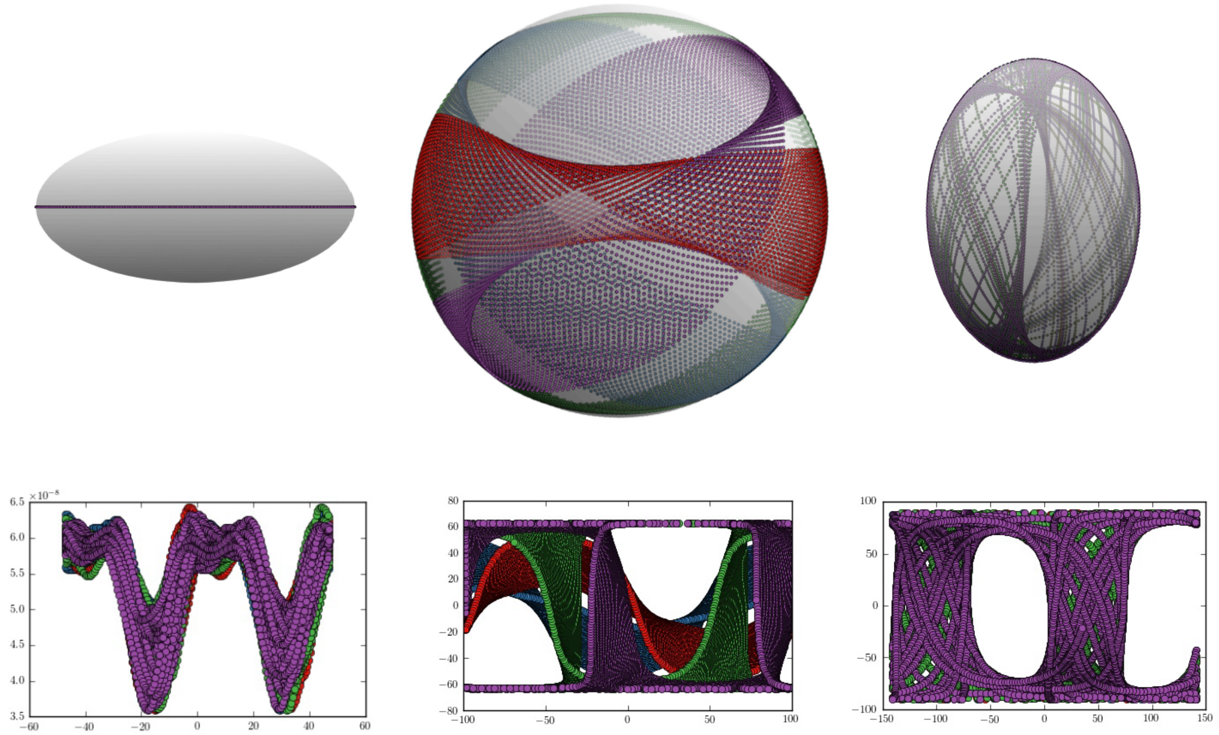

Figure 1: SI Active partcicle dynamics on spheroids: Trajectories of the four self-propelled particles, each color corresponds to one particle, shown on an oblate spheroid, the sphere and a prolate spheroid (from left to right), together with the Gall-Peters projection. The results for the sphere correspond to Figure 2b) bottom. The results for oblate and prolate spheroids significantly differ from Figure 3b) and a), respectively.
